# Using Modified Technology Acceptance Model to Evaluate the Adoption of a Proposed IoT-Based Indoor Disaster Management Software Tool by Rescue Workers

**DOI:** 10.3390/s22051866

**Published:** 2022-02-26

**Authors:** Preetinder Singh Brar, Babar Shah, Jaiteg Singh, Farman Ali, Daehan Kwak

**Affiliations:** 1 Chitkara University Institute of Engineering and Technology, Chitkara University, Rajpura 140401, Punjab, India; preetinder.brar@chitkara.edu.in; 2 College of Technological Innovation, Zayed University, Dubai 4783, United Arab Emirates; babar.shah@zu.ac.ae; 3 Department of Software, Sejong University, Seoul 05006, Korea; farmankanju@sejong.ac.kr; 4 School of Computer Science and Technology, Kean University, Union, NJ 07083, USA

**Keywords:** internet of things, IoT framework, indoor disaster management, technology acceptance model

## Abstract

Advancements in IoT technology have been instrumental in the design and implementation of various ubiquitous services. One such design activity was carried out by the authors of this paper, who proposed a novel cloud-centric IoT-based disaster management framework and developed a multimedia-based prototype that employed real-time geographical maps. The multimedia-based system can provide vital information on maps that can improve the planning and execution of evacuation tasks. This study was intended to explore the acceptance of the proposed technology by the specific set of users that could potentially lead to its adoption by rescue agencies for carrying out indoor rescue and evacuation operations. The novelty of this study lies in the concept that the acceptability of the proposed system was ascertained before the complete implementation of the system, which prevented potential losses of time and other resources. Based on the extended Technology Acceptance Model (TAM), we proposed a model included factors such as perceived usefulness, perceived ease of use, attitude, and behavioural intention. Other factors include trust in the proposed system, job relevance, and information requirement characteristics. Online survey data collected from the respondents were analyzed using structural equation modelling (SEM) revealed that although perceived ease of use and job relevance had significant impacts on perceived usefulness, trust had a somewhat milder impact on the same. The model also demonstrated a statistically moderate impact of trust and perceived ease of use on behavioural intention. All other relationships were statistically strong. Overall, all proposed relationships were supported, with the research model providing a better understanding of the perceptions of users towards the adoption of the proposed technology. This would be particularly useful while making decisions regarding the inclusion of various features during the industrial production of the proposed system.

## 1. Introduction

Earthquakes and other environmental hazards have remained the causes of numerous disaster events. Most of these disasters cause grave damage to life and property. A significant number of such disasters comprise building fires and building collapses.

In the past century, there have been tremendous enhancements in different specializations of engineering such as civil and structural engineering, electronics, and allied fields, and computer science engineering which has led to humans finding newer and better ways to tackle disaster situations. Buildings now offer better safety due to the use of earthquake-resistant structures and the deployment of fire-fighting equipment inside. However, disasters still strike at unexpected times, and damage to life and property continues to happen. In 2018, India alone witnessed 7887 incidents of building fires, both residential and commercial, that resulted in 7779 fatalities [1]. Similarly, there were 1953 cases of building/structure collapses in 2018 that claimed 2017 deaths [2]. In most cases, the loss of life can be attributed to the fact that the emergency response teams are mobilized after a considerable time lag due to the largely manual incident reporting systems

The availability of Internet of Things (IoT) devices and low-cost sensors for measuring different parameters such as humidity, temperature, pressure, and smoke density has paved the way for generating ubiquitous applications for a varied set of requirements. The market for the IoT has witnessed a sea-change in the last few years. In 2018, the global IoT market was valued at USD 190 billion. With the rapid adoption of IoT technology for various projects, it is projected that by 2026, the IoT market will be valued at USD 1102.6 billion [3]. The IoT-based applications have attracted voluminous attention and researchers, as well as engineers, have been exploring the possibility of using IoT for every possible scenario including disaster management [4,5,6,7,8].

In recent years, IoT technologies have drawn the attention of the Government of India (GoI). The GoI launched the National Smart Cities Mission with the intention to develop sustainable and resident-friendly smart cities throughout the country [9] by integrating a wide range of services into a single Information and Communication Technology (ICT) [10]. The benefits of the integrated services are more pronounced if they are automated through the implementation of IoT infrastructures [11]. IoT implementation in smart cities can provide an automatic rendering of services, such as vehicle parking, monitoring and rerouting of vehicular traffic, weather services, surveillance systems, and environmental pollution monitoring, thereby decreasing the time and human effort spent on those activities.

Emergency and rescue services are considered amongst the most important services provided by city administrations besides health care, public security, public works, etc. Efficient communication holds the key to the successful management and execution of disaster rescue operations [12]. Rescue teams need critical data regarding incidents for the better planning of rescue missions. However, since such data are often not available, rescuers are forced to rely on unstructured, ambiguous information that is received from different persons and is usually marred by uncertainty.

**Figure 1:**
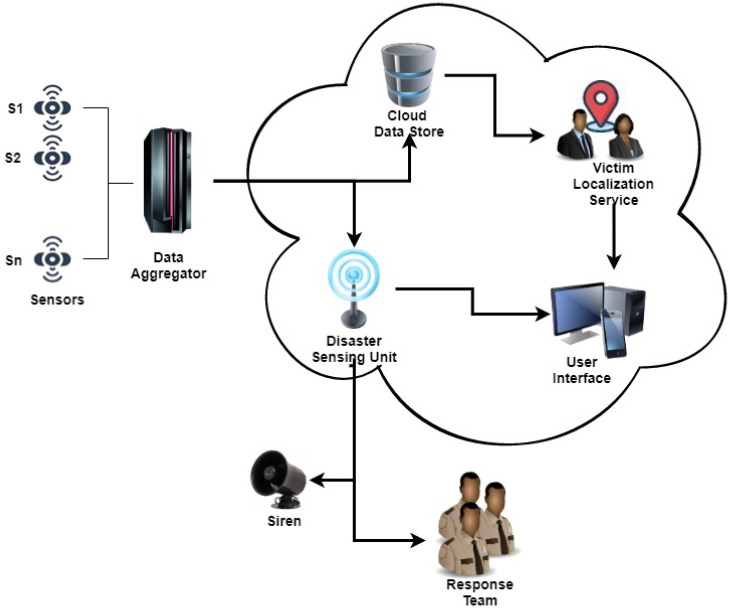
Proposed framework based on cloud-centric IoT-based for locating victims in indoor disasters.

With the IoT, it is possible to implement regular monitoring of buildings and to generate warnings or alerts at an early stage of any disaster-like situation. A cloud-centric IoT-based disaster management framework for indoor rescue operations was proposed by the authors in a previous study and is depicted in Figure 1 [13]. This framework allows for the automatic reporting of indoor disaster situations to emergency response teams without any human intervention. The reporting is done within a few seconds to all the stakeholders who are mapped to the disaster-struck building. The details of the exact location of the disaster (fire/building collapse) within the building are also reported. The framework also has provisions for reporting the count of probable victims inside the disaster-struck building, thereby allowing rescue/evacuation teams to effectively plan the evacuation operations en route to the site of the disaster. However, since such IoT-based systems are not reportedly deployed at present, most emergency and rescue team members are not aware of the benefits of systems based on such frameworks. Therefore, it is imperative that rescue team members become acquainted with this innovative technology to enable them to its adoption. We developed a prototype of the system to inform rescue team members about the proposed system. The prototype comprises the integration of Google Maps with locations of various sensors installed within a building. The count of persons at any location and the detection of disaster-like situations are depicted using heat maps. These details are dynamically fetched from a cloud database.

Various sensors such as fire sensors, smoke sensors, accelerometers, and person counters are installed at specific locations in large buildings. These sensors collect and supply necessary data to a specialized subsystem called a data aggregator (DA) that consolidates them into data packets, each bearing a unique timestamp. The data packets are transmitted over the cloud where the data are stored in an online repository called the cloud datastore (CD). This process is performed on a continuous basis for each set of sensor values received from different commercial facilities, and the data packets are stored in appropriate data buckets in the CD.

A module called the disaster-sensing unit (DSU) is installed as a service on the cloud. The data collected by DA are analyzed by the DSU to ascertain whether the disaster-like situation has arisen inside any of the buildings that are listed in the system’s database. In the event of an affirmative outcome, the DSU immediately activates the push notification (PN) module, which then sends alert messages to all stakeholders, including the floor supervisor and the response teams (RTs) that are stationed in the affected area, through SMS/email. All stakeholders are provided the necessary data by the victim localization service (VLS) via the user interface (UI).

The VLS module presents important data to the RTs through the UI, which displays real-time information via pre-configured indoor building maps. The heat maps are superimposed onto floor maps to show the count of victims trapped inside disaster-struck buildings. This information can prove helpful in launching rescue operations in areas where victims are present. The UI can be accessed by the RTs with any internet-enabled device. However, in the event of the internet connection between the sensors and the DA or the connection between the DA and the MB becoming non-functional, the VLS presents the most recent snapshot of the available data.

## 2. Methodology

### 2.1. Tools and Techniques Employed

The authors of this study employed the Technology Acceptance Model (TAM) to establish the willingness of rescue team members to adopt the proposed systems. Proposed by Davis in 1986 as an outcome of his research work [14], TAM is extensively used to generate models to reflect the intentions of the intended users to accept and use the new technology [15,16,17]. An extended version of the technique was later proposed in 2000 and is known as TAM2 [15].

This study intends to ascertain the level of acceptability of the proposed IoT-based indoor disaster management software tool by rescue workers. Since this system is not available commercially and is not yet deployed in any building, therefore a custom-built simulator was deployed to imitate the flow of persons in the indoor environment of any large commercial complex by synthetically modulating the virtual sensor values. The simulator was developed using the Java programming language and was deployed on Google Cloud as a service. A web-based user interface (UI) was also developed using the Google Maps API and the simulated situational data was displayed by overlaying the heat maps on Google Maps. The UI provided ready reference to the rescue teams and could be accessed using any smart device.

This study employed structural equation modelling (SEM) for analysis because of its capability to test the hypothesized relationships established in a research model with multiple constructs. It also considers analysis error terms that cannot be uncovered using other prominent techniques such as multiple regression [18]. Data were collected with a structured questionnaire, the collected responses of which were analyzed using IBM AMOS 20.

### 2.2. Literature Collection

A manual search was performed in the Google Scholar database to access the literature related to TAM. Initially, an attempt was made to look out for the articles published in the recent past between 2011 and 2020. Since the TAM is implemented and evaluated through the SEM technique, articles related to SEM were also queried. The keywords used to search the articles were “Technology Acceptance Model” OR “Acceptance of New Technology” OR “Technology Acceptance Framework” OR “Technology Acceptance in Disaster Management” OR “Adoption of Emergency Rescue Technology using TAM” OR “Structural Equation Modelling in Technology Acceptance Model”. A total of 892 articles were shortlisted in the first phase. The Mendeley desktop application was used to maintain the repository of downloaded articles. Then, a manual screening process was initiated for the selection of relevant literature. It was observed that the literature cited by most of the articles pointed to the articles published from the late 1980s to the early 2000s. This was the period in which the TAM was proposed and evolved into TAM2. Therefore, the articles by the original proposers and the early implementors of the TAM were also included in the search. In addition to the TAM, articles on SEM were also queried. The absolute number of records after this step was 912 articles.

Having formed a repository, we initiated the screening phase using Mendeley, wherein we attempted to remove any duplicate records. This was followed by the manual examination of the titles and abstracts of the articles with the intent to determine relevance to the present study. This step removed most irrelevant articles, and only 187 records were found suitable for the study.

Since the TAM is extensively used by the researchers working in the domain of technology acceptance, in this study, only the articles that received significant recognition by subsequent research (with some articles having more than 8000 citations according to Google Scholar) were considered. Finally, 91 articles were shortlisted for inclusion in the article.

## 3. Literature Review and Development of Research Model

Models that have been employed in research on technology adoption have considered people’s ideas, considerations, and attitudes regarding certain technologies. One of the most prominent models is the TAM, which mainly considers two constructs: perceived usefulness (PU) and perceived ease of use (PEU) [14]. Various studies have extended the Technology Acceptance Model to validate respective research models [15,19,20]. The authors of this research work considered prominent constructs of the TAM, namely PU, PEU, attitude towards adoption (ATA), and behavioural intention to use (BI). Additionally, some new constructs were introduced in the extended version of the TAM, and since the proposed system is to be used during emergency situations, it is imperative that the factors of trust (TR), job relevance (JR) and information requirement characteristics (IRC) regarding the acceptance of the proposed system be evaluated in conjunction with the conventional constructs.

**Figure 2:**
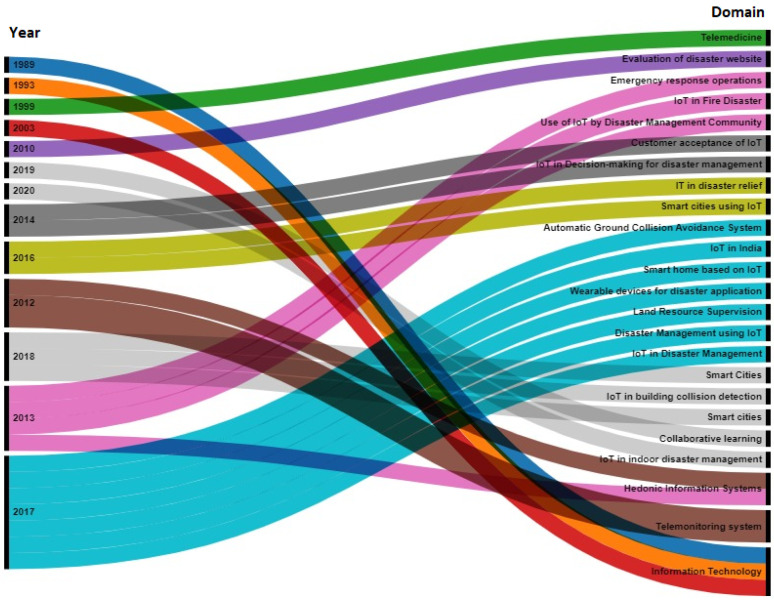
Alluvial diagram depicting the trend of research using TAM.

The study of related literature revealed that the use of IoT in disaster management has been suggested previously. However, a study related to acceptance of a complete IoT-based framework for disaster management thorough advanced statistical techniques had not been conducted. Figure 2 depicts the advancements in research trends in this domain.

### 3.1. Perceived Ease of Use

Studies on the original and extended TAM have revealed that users perceive a technology to be useful if it is user-friendly and easy to use. The ease of use refers to how easy the technology is to use/how much effort is required. Thus, the ease of use has a strong bearing on perceptions of usefulness [14,15,16,21,22,23,24]. Furthermore, TAM depends on the perception that ease of use influences the intended user’s intention to use a technology [14,15,16,23,24,25,26,27,28]. Therefore, the following hypotheses were derived.

**Hypothesis** **1** **(H1).** *A significant relationship exists between the PEU and the PU*.

**Hypothesis** **2** **(H2).** *A significant relationship exists between the PEU and BI*.

### 3.2. Perceived Ease of Use

Perceived usefulness is the user’s perception of the ability of the new technology to improve job performance. A statistically significant relationship between the perceived usefulness and attitude towards adoption of technology has been reported in previous studies [29,30]. On the same lines, the authors of this study perceived that a positive attitude towards the proposed technology may be reflected by the intended users if they find it useful. Thus, the following hypothesis was derived.

**Hypothesis** **3** **(H3).** *A significant relationship exists between PU and ATA*.

### 3.3. Attitude towards Adoption

Previous studies have shown that a user’s attitude towards a technology affects their intention to use it [31,32,33,34]. It has also been shown that even if the use of an innovative technology is mandatory, there may be certain users who do not wholeheartedly accept it, which may hamper implementation [35,36].

**Hypothesis** **4** **(H4).** *A significant relationship exists between ATA and BI*.

### 3.4. Job Relevance

The proposed system is intended to be primarily used by rescue workers who specialize in such operations [37] and can relate the usefulness of the proposed system in executing their job-related activities.

In this regard, Venkatesh [15,27] considered job relevance as a cognitive factor in the extended TAM. This allowed them to measure the perception of the individuals regarding the usage of technology in their jobs. Other studies have also been carried out to ascertain the effect of job relevance on perceived usefulness [16,38]. The following hypotheses were accordingly derived.

**Hypothesis** **5** **(H5).** *A significant relationship exists between JR and PU*.

**Hypothesis** **6** **(H6).** *A significant relationship exists between JR and BI*.

### 3.5. Trust in Proposed System

As new technologies are introduced in any domain, users may experience some uncertainty. Accordingly, the authors of some studies have considered trust in technology as an important factor [39,40,41,42]. Since the proposed system in this study is a disaster management tool for obtaining important data to enable effective evacuation in case of an indoor emergency, we also considered trust in the proposed system because it could have a direct bearing on the perceptions of rescue team members towards the system. It has been shown in previous research that trust has an impact on perceived usefulness and behavioural intention to use new technologies [17,42]. Thus, for this study, it was hypothesized that:

**Hypothesis** **7** **(H7).** *A significant relationship exists between TR and PU*.

**Hypothesis** **8** **(H8).** *A significant relationship exists between TR and BI*.

### 3.6. Information Requirement Characteristics

Any rescue operation requires good planning and appropriate resource allocation. Since the proposed system was designed to be mainly used in life-threatening conditions, it is expected that it should be able to provide correct and relevant information to rescue team members to enable them to act accordingly. In this regard, it is considered that if correct and relevant information is available, then the users will believe that the system is relevant to their job and will develop trust in the system. Therefore, the following hypotheses were derived.

**Hypothesis** **9** **(H9).** *A significant relationship exists between IRC and JR*.

**Hypothesis** **10** **(H10).** *A significant relationship exists between IRC and TR*.

**Figure 3:**
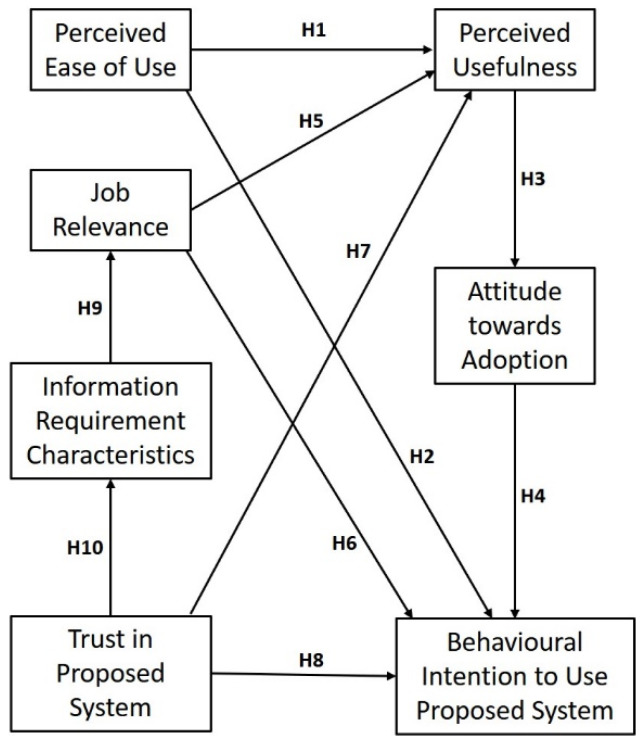
Research model for the proposed indoor disaster management system.

Figure 3 depicts the extended TAM research model based on the hypotheses defined above.

## 4. Research Methodology

### 4.1. Measurement Scales

The authors of this study used an online structured questionnaire to collect data. The various indicators were based on extended TAM constructs, as shown in Figure 3. The questionnaire adapted the scale items for different constructs from measures that were previously validated in earlier studies. The indicators for each measurement model were then restated to align with the characteristics of proposed indoor disaster management system. The scales for PEU, PU, JR, and BI were adapted from [15]. Items relating to attitude towards adoption (ATA) were adapted from [22]. The items for trust (TR) were adapted from [17], and the items for information requirement characteristics (IRC) were adapted from [43].

### 4.2. Content Validity

The online questionnaire carried 24 indicators conforming to the Technology Acceptance Model, as well as 9 demographic questions. The questionnaire was shared with experts to validate that the various indicators represent the specific latent variables. On the recommendation of the experts, one indicator that was considered redundant was dropped from the questionnaire. This resulted in 23 indicators remaining in the questionnaire. All indicators were designed to be answered on a seven-point Likert scale in which a value of 1 indicated “strongly disagree” and a value of 7 indicated “strongly agree”.

In order to ensure the easy access and submission of replies by users, we initiated a pilot study wherein 30 security staff members were sent a link to the questionnaire. The pilot study did not yield any negative issues except for requests to reframe one question for better understandability and to provide all questions in Devanagari (Hindi) script in addition to English. The feedback was duly acknowledged, and the questionnaire was set as a bilingual instrument. The English version of the questionnaire is presented in Table 1. The responses recorded during the pilot study were subjected to Cronbach’s alpha using SPSS 21 to measure internal consistency. With Cronbach’s alpha coefficients being computed above 0.70 for each of the constructs, all initial scales employed in the research model were found to be reliable measures for their respective constructs.

### 4.3. Data Collection

The link of the online questionnaire was shared with two different categories of respondents. The first category comprised the full-time fire fighters deployed in the three different cities of the state of Punjab (India)—SAS Nagar, Zirakpur and Derabassi—and three fire stations of Chandigarh. This category of respondents is extensively trained to perform evacuation operations in case of indoor fire or building collapse. The second category of respondents comprised the security staff of large shopping malls and buildings with various corporate offices. The respondents belonging to this category are also trained to respond to the disaster-like situations within the building and are usually the first to respond to any mishap. However, those of category 1 are equipped with specialized equipment for fire-fighting operations and evacuation. Police personnel are also trained for carrying out rescue and evacuation operations, though they are not directly involved. Therefore, police personnel were also included in the scope of the study and were placed in category 2.

To allow the respondents a better understanding of the proposed IoT-based system for disaster management, we felt the need to provide a demonstration of the system to rescue staff. Therefore, we contacted fire station officers, who facilitated meetings between the authors and the category 1 respondents. Similarly, the security and safety supervisors of the commercial complexes facilitated meetings with the category 2 respondents. The demonstration of the system was followed by the sharing of the link to the online questionnaire.

Having received 270 responses, a data pre-processing was initiated to remove duplicate responses. Only one response sheet from each respondent was used for analysis, and the duplicate responses were considered invalid. No case of incomplete data was observed because all questions in the online questionnaire were mandatory. Finally, only 257 responses were treated as valid. From about 180 fire fighters deployed in the fire stations visited by the authors, only 106 participated in the survey. All other 151 responses were filled by the second category of respondents.

**Figure 4:**
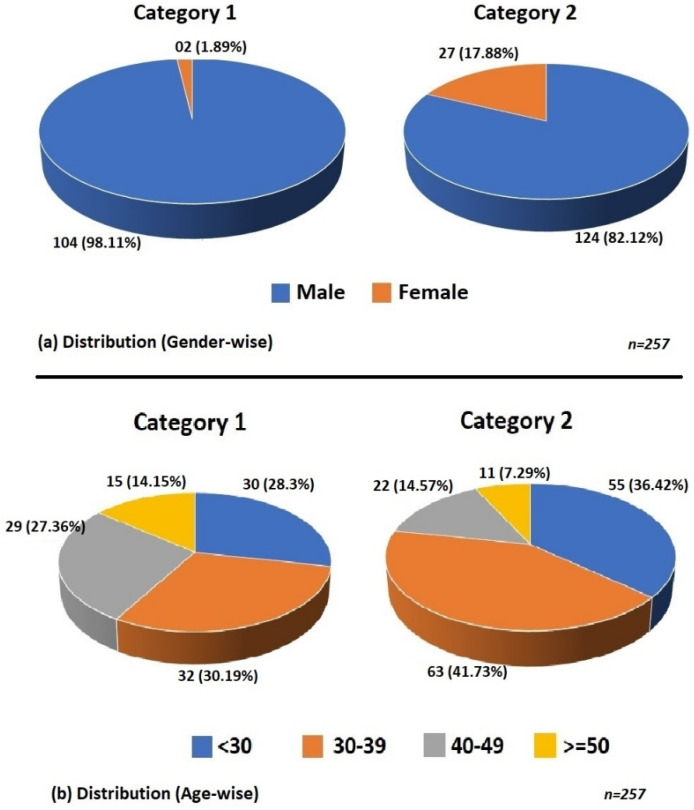
Demographic characteristics of rescue team members.

The demographic characteristics of the respondents are shown in Figure 4. As shown in Figure 4a, a major percentage of responders were male, which can be attributed to the hazardous nature of the job. As shown in Figure 4b, 33.07% of overall respondents were younger than 30 years old and 36.97% belonged to age group of 30–39 years. Only 10.12% of the respondents were older than 50 years old.

## 5. Data Analysis and Results

### 5.1. Descriptive Statistics

In order to determine the mean and standard deviation (SD) for the various responses recorded, descriptive statistical tests were conducted. The results of descriptive statistics are presented in Table 2. All mean values were found to be more than 5.90, which indicated that the respondents had positive perceptions for the proposed system. Table 3 depicts the correlation matrix for all constructs defined in the research model. All constructs were found to be positively correlated.

### 5.2. Measurement Model Analysis

The causal paths defined with the various hypotheses were tested using the SEM technique, which has become popular in the research community for estimating causal relations via amalgamations of statistical data with qualitative causal assumptions. In this study, SEM was conducted using AMOS 20.

#### 5.2.1. Confirmatory Factor Analysis

Confirmatory factor analysis (CFA) is a multivariate statistical technique that is popular among researchers for measuring the reliability and validity of constructs. The overall goodness-of-fit indices of this study supported an acceptable overall model fit, with χ^2^/df = 1.555; GFI = 0.905; CFI = 0.961; AGFI = 0.875; IFI = 0.961; TLI = 0.952; NFI = 0.898; and RMSEA = 0.047. Therefore, the measurement model matched the structure of the proposed research model.

#### 5.2.2. Convergent Validity

Factor loading implies the correlation between an original variable and a derived factor. If all standardized item factor loadings are found to be more than 0.70, then convergent validity is confirmed. The factor loadings of various latent variables with their respective indicators and the other computed measures for the assessment of convergent and discriminant reliability in this study are shown in Table 4. All factor loadings (standardized) in CFA were observed to be more than 0.70. Additionally, the average variance extracted (AVE) values were computed and found to be more than 0.50 [44,45]. The composite reliability (CR) of all constructs was computed and found to be above 0.767 (the recommended cut-off is 0.70) [44,45,46]. Finally, the minimum value of the Cronbach’s alpha was found to be 0.765 (the recommended cut-off is 0.70) [44]. These results clearly support the convergent validity.

#### 5.2.3. Discriminant Validity

The extent to which a construct is truly distinct from the other constructs of the structural model is called discriminant validity. A model is said to conform to discriminant validity if (i) the maximum shared variance (MSV) is less than average shared variance (ASV) and (ii) the ASV is less than the AVE. Both of these conditions were met by the proposed model, as is shown in Table 4. Other than this, the square root values of all AVE were computed and observed to be higher than the inter-construct correlations [47], thereby confirming the good discriminant validity.

## 6. Results—Hypothesis Testing

The structural model helped explain the relationship amongst the constructs defined in the research model with sound theoretical background. The various hypotheses, as depicted in the research model shown in Figure 3, were tested using the SEM technique. The results of hypotheses testing are demonstrated in Table 5. The fit indices—χ2/df = 2.078; GFI = 0.870; AGFI = 0.837; CFI = 0.920; TLI = 0.907; IFI = 0.920; NFI = 0.857; and RMSEA = 0.065—indicated that the dataset was acceptable [48].

The H1, H3, H4, H5, H6, H9 and H10 hypotheses were found to be highly significant. As also reported in previous research, this study recorded the significant impact of perceived ease of use on perceived usefulness (β = 0.265; t = 4.329; *p* < 0.001). Therefore, hypothesis H1 was supported. The perceived ease of use was also found to have an impact on behavioural intention (β = 0.183; t = 2.784; *p* < 0.010), thereby confirming hypothesis H2.

The perceived usefulness had a significant impact on attitude towards adoption (β = 0.612; t = 5.671; *p* < 0.001), thus confirming hypothesis H3. The attitude towards adoption was further found to have a significant impact on behavioural intention (β = 0.191; t = 3.717; *p* < 0.001), so hypothesis H4 was supported.

Job relevance was found to have significant relationship with perceived usefulness (β = 0.295; t = 5.148; *p* < 0.001) and behavioural intention (β = 0.381; t = 5.717; *p* < 0.001), thus supporting hypotheses H5 and H6. Hypotheses H9 and H10 examined the effect of information requirement characteristics on job relevance and trust in the proposed system. The information requirement characteristics was found to have a significant relationship with job relevance (β = 0.683; t = 8.078; *p* < 0.001) and trust (β = 0.464; t = 6.272; *p* < 0.001). Therefore, hypotheses H9 and H10 were supported at the 0.001 level of significance.

Trust was observed to be related to perceived usefulness (β = 0.164; t = 2.548; *p* < 0.05) and behavioural intention (β = 0.179; t = 2.492; *p* < 0.05), thus supporting hypotheses H7 and H8.

Thus, this study revealed support for all hypotheses H1–H10.

## 7. Discussion and Implications

This study was intended to find if rescue and emergency workers had any inclination towards using the proposed technology for disaster management. Based on previous studies, some additional construct—namely, job relevance, trust in proposed system and information requirement characteristics—were used to form the research model.

We developed a prototype of the proposed system that provided a first-hand experience of the final system. The weblink of the prototype deployed on the cloud was shared with respondents who were able to use it on their internet-enabled smartphones to experience the system in use.

Since the proposed disaster management system is intended to be used by rescue workers, it is important that they perceive the system to be job-relevant and trust-worthy. These factors were found to have a direct impact on perceived usefulness and behavioural intention to use the system. The information requirement characteristics also reflected a significant relationship with job relevance and trust in proposed system. This implies that if the important situational information inside an affected building is available, then a sense of trust in the system is developed by users. This further leads to better perceived usefulness and behavioural intention. Providing situational information using IoT devices can enable users to plan and initiate rescue operations from a point within the building where the probability of finding victims is high, thereby improving the chances of saving more lives in the event of an indoor disasters such as building fire or partial collapse. With the clear establishment of relationships between the vital constructs, it is expected that the final version of the system, with the implementation of actual sensors, will find better acceptability among rescue teams. Furthermore, the authors of this study were able to establish a clear understanding regarding whether the proposed system can be commercially produced. In view of the positive responses of the intended users towards the proposed system, it is inferred that systems based on the framework can be manufactured at the commercial level and be implemented in various large commercial establishments.

## 8. Limitations of the Study

This was a cross-sectional study based on a convenient sampling method that was conducted in selected fire stations and commercial facilities of a particular region. According, our results may not be generalized. Furthermore, the amount of responses received from female respondents was quite low since there are very few women in the region who seek employment in rescue and security services. Additionally, the proposed IoT-based disaster management system is dependent on internet. In the event of the internet becoming unavailable during the disaster situation, the live situational data inside the buildings cannot be made available to rescue teams. However, the system can still provide the most recent snapshot of the situational data that can enable rescue teams to plan rescue operations.

## Figures and Tables

**Table 1:** Measurement scales.

TAM Latent Variable	Indicator
Perceived Usefulness (PU)	[PU01]—The proposed system can improve the performance of rescue teams. [PU02]—The proposed system can improve the effectiveness of rescue operations. [PU03]—The proposed system will be useful to you as a rescue worker. [PU04]—The proposed system can improve the productivity of rescue operations.
Perceived Ease of Use (PEU)	[PEU01]—The proposed system can be used in easy manner. [PEU02]—The data provided by the proposed system do not require a much effort to understand. [PEU03]—Your communications with the proposed system will be simple and understandable.
Attitude towards Adoption (ATA)	[ATA01]—Adopting the proposed system for indoor rescue operations is a good idea. [ATA02]—The proposed system may be beneficial to you. [ATA03]—You have positive opinion about using the proposed system.
Behavioural Intention to Use (BI)	[BI01]—You are willing to voluntarily use the proposed system. [BI02]—If you get the proposed system, you will to use it during rescue operations. [BI03]—You would make full use of the proposed system.
Trust in Proposed System (TR)	[TR01]—The proposed system can provide trustworthy information for rescue operations. [TR02]—You think that the information will be more reliable if it is collected by the proposed system than if it is collected manually. [TR03]—As a rescue team member, you can trust the information provided by the proposed sensor-based system.
Job Relevance (JR)	[JR01]—The proposed system is relevant to your job involving rescue operations. [JR02]—The proposed system is important for your job as a rescue team member. [JR03]—The proposed system is appropriate for your job due to its occupational hazards.
Information Requirement Characteristics (IRC)	[IRC01]—Rescue teams do not get appropriate situational information from inside the disaster-struck building. [IRC02]—Rescue teams are generally unaware of the count of people stuck in disaster-struck building. [IRC03]—Better decisions can be made if accurate situational awareness exists. [IRC04]—The resource allocation for disaster response is usually not optimal since situational information inside a building is not available.

**Table 2:** Descriptive statistics.

Construct	Mean	Std. Deviation
PEU	6.010	0.831
PU	6.101	0.793
ATA	5.901	0.905
BI	6.042	0.818
TR	5.944	0.824
JR	6.043	0.851
IRC	6.331	0.772

Note: *N* = 257.

**Table 3:** Correlation matrix.

		1	2	3	4	5	6	7
1	PEU	1						
2	PU	0.396 **	1					
3	ATA	0.262 **	0.353 **	1				
4	BI	0.360 **	0.372 **	0.417 **	1			
5	TR	0.319 **	0.377 **	0.399 **	0.442 **	1		
6	JR	0.337 **	0.454 **	0.359 **	0.523 **	0.535 **	1	
7	IRC	0.314 **	0.385 **	0.293 **	0.484 **	0.372 **	0.469 **	1

Note: *N* = 257; * *p* < 0.05, ** *p* < 0.01.

**Table 4:** Reliability measures for convergent and discriminant validity.

Construct	Indicator	Factor Loading	Cronbach’s Alpha	CR	AVE	MSV	ASV	Sqrt (AVE)
PEU	PEU01	0.722	0.822	0.823	0.608	0.221	0.163	0.780
	PEU02	0.833						
	PEU03	0.780						
PU	PU01	0.747	0.846	0.850	0.588	0.286	0.211	0.767
	PU02	0.852						
	PU03	0.724						
	PU04	0.737						
ATA	ATA01	0.854	0.868	0.869	0.688	0.263	0.170	0.829
	ATA02	0.824						
	ATA03	0.810						
BI	BI01	0.742	0.765	0.767	0.523	0.426	0.290	0.723
	BI02	0.705						
	BI03	0.722						
TR	TR01	0.790	0.815	0.817	0.599	0.432	0.246	0.774
	TR02	0.712						
	TR03	0.816						
JR	JR01	0.820	0.826	0.827	0.615	0.432	0.298	0.784
	JR02	0.763						
	JR03	0.769						
IRC	IRC01	0.785	0.885	0.886	0.660	0.338	0.213	0.812
	IRC02	0.793						
	IRC03	0.832						
	IRC04	0.839						

**Table 5:** Hypothesis testing results.

H	Relationship	Estimate	S.E.	C.R.	*p*	Result
H1	PU ← PEU	0.265	0.061	4.329	0.000	Supported
H2	BI ← PEU	0.183	0.066	2.784	0.005	Supported
H3	ATA ← PU	0.612	0.108	5.671	0.000	Supported
H4	BI ← ATA	0.191	0.053	3.617	0.000	Supported
H5	PU ← JR	0.295	0.057	5.148	0.000	Supported
H6	BI ← JR	0.381	0.067	5.717	0.000	Supported
H7	PU ← TR	0.164	0.064	2.548	0.011	Supported
H8	BI ← TR	0.179	0.072	2.492	0.013	Supported
H9	JR ← IRC	0.683	0.085	8.078	0.000	Supported
H10	TR ← IRC	0.464	0.074	6.272	0.000	Supported

## Data Availability

Not Applicable.
